# Supplementary material for: Profiling host ANP32A splicing landscapes to predict influenza A virus polymerase adaptation
Source: Nat Commun. 2019 Jul 30;10:3396. doi: 10.1038/s41467-019-11388-2 (PMC6667478; doi:10.1038/s41467-019-11388-2)
Supplement: Supplementary file 1 — Supplementary Information [file 41467_2019_11388_MOESM1_ESM.pdf]

Supplementary Information

Profiling Host ANP32A Splicing Landscapes to Predict Influenza A Virus Polymerase Adaptation

Domingues *et al*.

Corresponding author: Benjamin G. Hale
Email: hale.ben@virology.uzh.ch

This PDF file includes:

Supplementary Figures 1-3
Supplementary Tables 1-4

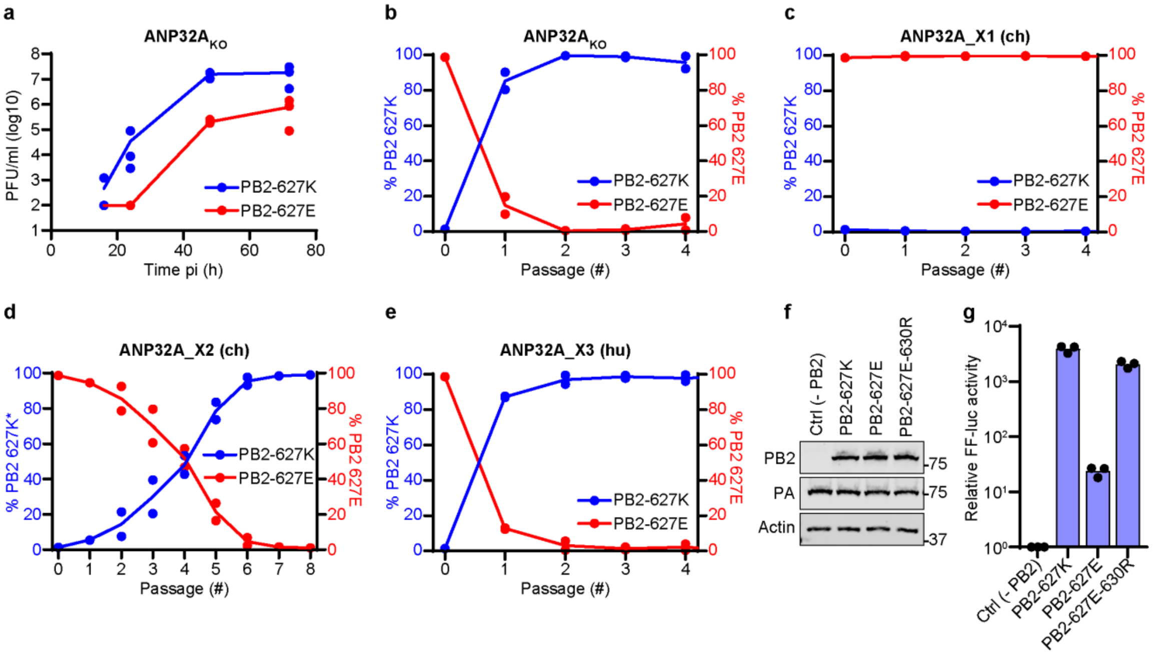

Supplementary Figure 1. ANP32A variants differentially impact avian-signature IAV replication and selection. (**a**) Viral growth kinetics of rWSN-based viruses expressing PB2-627E or PB2-627K in A549-ANP32A$_{KO}$ cells (MOI = 0.001 PFU/cell). Mean values from three independent experiments are plotted, with the individual data points shown. (**b-e**) Competition assays between rWSN-based viruses expressing PB2-627E or PB2-627K (99:1 input ratio) in the indicated cell-lines. P0 represents input. Percentage of PB2-627K/E was determined by NGS. Mean values from two independent experiments are plotted, with the individual data points shown. *Note, for panel d, levels of the compensatory PB2-630R mutation were also determined and included together with PB2-627K. (**f**) Western blot analysis of lysates from human 293T cells transfected with the indicated vPol constructs. (**g**) Polymerase reconstitution assay assessing the ability of PB2-630R to compensate for PB2-627E vPol activity in human 293T cells. Bars represent mean values from three independent experiments, with the individual data points shown. Source data for all panels are provided in the Source Data file.

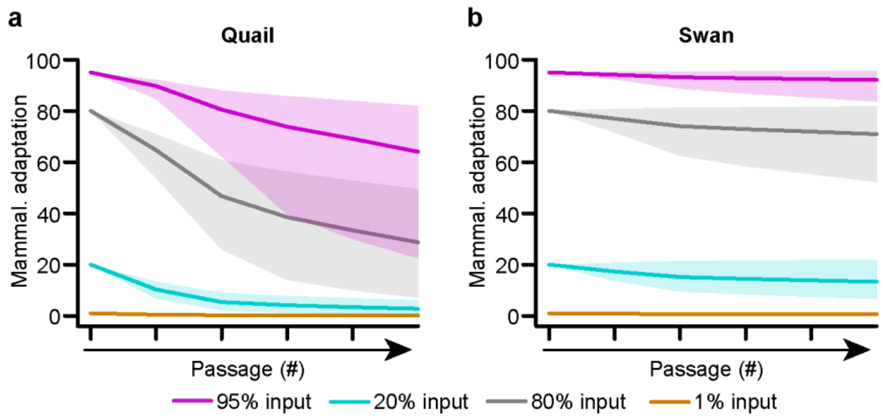

Supplementary Figure 2. Modeling the impact of species' ANP32A splice variant ratios on selection of mammalian- or avian-like IAV adaptations. (**a-b**) Model predictions of mammalian-like vPol substitution selection in the indicated species, taking into account their ANP32A splice variant ratios as shown in Figure 3a. Each input mammalian-like vPol percentage assessed is indicated by a separate color. The solid lines indicate the model prediction with the best estimates of virus production rates, and the lighter areas show the 95% bootstrap confidence intervals.

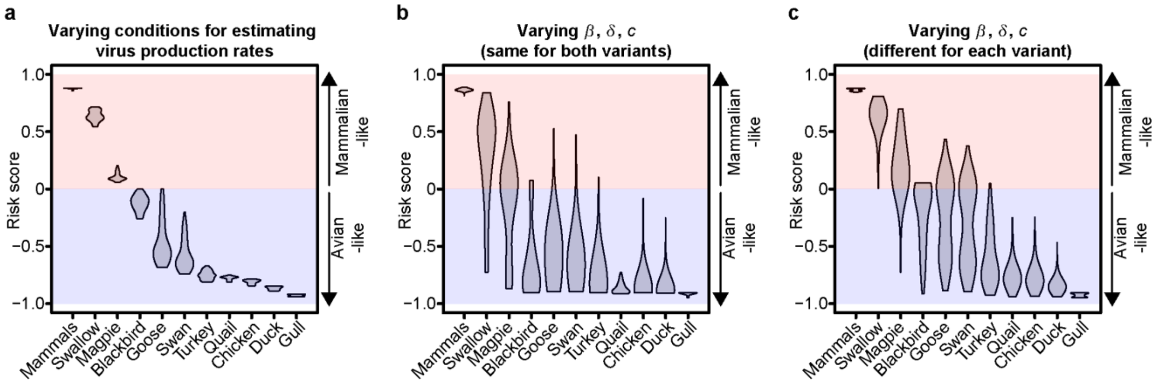

Supplementary Figure 3. Sensitivity analyses of the model to predict the impact of ANP32A splice variant ratio on selection of mammalian-like IAV adaptations. (**a-c**) Violin plots of the estimated relative risk scores for each indicated species to select for avian- or mammalian-signature vPol adaptations: (**a**) varying conditions for estimating virus production rates; (**b**) varying infection rate (β), rate of infected cell death (δ), and rate of viral clearance (c) [same for both variants]; (**c**) varying infection rate (β), rate of infected cell death (δ), and rate of viral clearance (c) [different for each variant].

Supplementary Table 1

Summary of ANP32A transcript analyses from cells of different species

**Species (cell-line)**	**aa sequence**	**ANP32A transcript (% ± SD)**
Human (A549 cultured at 33° or 37°C; BEPC; NEPC; SAEPC or HTEpC)	GYDRDDKEAPDSDAEGYVEGLDDEEEDEDEEEYDEDAQVVEDEEDED	X3-like (100%)
Pig (PK-15)[1]	GYDRDDKEASDSDAEGYVEGLDDDEEDEDEEEYDEDAQVVEDEEDEE	X3-like (100%)
Pig (NSK)	GYDRDDKEASDSDAEGYVEGLDDDEEDEDEEEYDEDAQVVEDEEDEE	X3-like (100%)
Dog (MDCK)[2]	GYDRDDKEAPDSDAEGYVEGLDDDEEDEDEEEYDEDAQVVEDEEDEE	X3-like (100%)
Mouse (MEFs)	GYDRDNKEAPDSDVEGYVEDDDEEDEDEEEYDEYAQLVEDEEEEDEE	X3-like (100%)
Bat (PaKi)	GYDRDDKEAPDSDAEGYVEGLDDDEEDEDEEEYDEDAQVVEDEEDEEEE	X3-like (100%)
Horse (NBL6)	GYDRDDKEAPDSDAEGYVEGLDDDEEDEDEEEYDEDAQVVEDEEDEE	X3-like (90 ± 2.3%)
Horse (NBL6)	*GYDRDDKEAPDSDAEGYVEGLDDDEEDVSGEEEEDEEGYNDGEVDRS*	*Other* (10 ± 2.3%*)*
Chicken (DF-1)	GYDRDDKEAPDSDAEGYVEGLDDEEEDEDVLSLVKDRDDKEAPDSDAEG	X1-like (66.0 ± 0.3 %)
Chicken (DF-1)	GYDRDDKEAPDSDAEGYVEGLDDEEEDEDVKDRDDKEAPDSDAEGYVEG	X2-like (24.9 ± 0.5%)
Chicken (DF-1)	GYDRDDKEAPDSDAEGYVEGLDDEEEDEDEEEYDDDAQVVEDEED	X3-like (9.1 ± 0.8%)
Chicken (CAM[3])	GYDRDDKEAPDSDAEGYVEGLDDEEEDEDVLSLVKDRDDKEAPDSDAEG	X1-like (65.2 ± 1.6 %)
Chicken (CAM)	GYDRDDKEAPDSDAEGYVEGLDDEEEDEDVKDRDDKEAPDSDAEGYVEG	X2-like (26.2 ± 2.3%)
Chicken (CAM)	GYDRDDKEAPDSDAEGYVEGLDDEEEDEDEEEYDDDAQVVEDEED	X3-like (8.6 ± 0.8%)

---

[1] ANP32A transcripts are identical between the porcine PK-15 and NSK cell lines.

[2] Dog (MDCK) sample presents two ANP32A variants coding for identical proteins (underlined, the synonymous, t→c, SNP).

clANP32A_A:

tatctcgatggctacgaccgggacgacaaggaggcccccgactcggacgctgagggctacgtggagggcctggatgacgacgaggaggacgagga tgaagaagaatatgatgaagatgctcaggtagtggaagacgaggaggatgaagagg

clanp32a_b:

tatctcgatggctacgaccgggacgacaaggaggcccccgactcggacgccgagggctacgtggagggcctggatgacgacgaggaggacgagg atgaagaagaatatgatgaagatgctcaggtagtggaagacgaggaggatgaagagg

[3] CAM, chorioallantoic membrane (n = 3).

Species (cell-line)	aa sequence	ANP32A transcript (% ± SD)
Chicken (LMH-1)	GYDRDDKEAPDSDAEGYVEGLDDEEEDEDVLSLVKDRDDKEAPDSDAEG	X1-like (62.6 ± 1.2 %)
Chicken (LMH-1)	GYDRDDKEAPDSDAEGYVEGLDDEEEDEDVKDRDDKEAPDSDAEGYVEG	X2-like (26.7 ± 0.3%)
Chicken (LMH-1)	GYDRDDKEAPDSDAEGYVEGLDDEEEDEDEEEYDDDAQVVEDEED	X3-like (10.8 ± 1.1%)
Chicken (SL-29)	GYDRDDKEAPDSDAEGYVEGLDDEEEDEDVLSLVKDRDDKEAPDSDAEG	X1-like (62.0 ± 1.5 %)
Chicken (SL-29)	GYDRDDKEAPDSDAEGYVEGLDDEEEDEDVKDRDDKEAPDSDAEGYVEG	X2-like (28.5 ± 1.3%)
Chicken (SL-29)	GYDRDDKEAPDSDAEGYVEGLDDEEEDEDEEEYDDDAQVVEDEED	X3-like (9.6 ± 1.3%)
Duck (CCL-141)	GYDRDDKEAPDSDAEGYVEGLDDEEEDEDVLSLVKDRDDKEA	X1-like (76.7 ± 1.3%)
Duck (CCL-141)	GYDRDDKEAPDSDAEGYVEGLDDEEEDEDVKDRDDKEAPDSDAE	X2-like (19.4 ± 1.6%)
Duck (CCL-141)	GYDRDDKEAPDSDAEGYVEGLDDEEEDEDEEEYDDDAQDR	X3-like (3.9 ± 0.3%)
Mallard (SEF-R)	GYDRDDKEAPDSDAEGYVEGLDDEEEDEDVLSLVKDRDDKEAP	X1-like (82.9 ± 1.5%)
Mallard (SEF-R)	GYDRDDKEAPDSDAEGYVEGLDDEEEDEDVKDRDDKEAPDSDAE	X2-like (15.4 ± 1.6%)
Mallard (SEF-R)	GYDRDDKEAPDSDAEGYVEGLDDEEEDEDEEEYDDDAQDR	X3-like (1.7 ± 0.2%)
Turkey (PF-R)	GYDRDDKEAPDSDAEGYVEGLDDEEEDEDVLSLVKDRDDKEAPDSDAEG	X1-like (67.9 ± 1.7%)
Turkey (PF-R)	GYDRDDKEAPDSDAEGYVEGLDDEEEDEDVKDRDDKEAPDSDAEGYVEG	X2-like (23.1 ± 1.3%)
Turkey (PF-R)	GYDRDDKEAPDSDAEGYVEGLDDEEEDEDEEEYDDDAQVVEDEEDR	X3-like (9.0 ± 1.7%)
Quail (QT-6)	GYDRDDKEAPDSDAEGYVEGLDDEEEDEDVLSLVKDRDDKEAPDSDA	X1-like (61.9 ± 0.7%)
Quail (QT-6)	GYDRDDKEAPDSDAEGYVEGLDDEEEDEDVKDRDDKEAPDSDAEGYV	X2-like (38.1 ± 0.7%)
Gull (SMF-R)	GYDRDDKEAPDSDAEGYVEGLDDEEEDEDVLSLVKDRDDKEAPDSDAEG	X1-like (100%)
Goose (GF-R)[4]	GYDRDDKEAPDSDAEGYVEGLDDEEEDEDVLSLVKDRDDKEAPDSDAEG	X1-like (67.7 ± 1.3%)
Goose (GF-R)	GYDRDDKEAPDSDAEGYVEGLDDEEEDEDVKDRDDKEAPDSDAEGYVEG	X2-like (6.9 ± 0.6%)
Goose (GF-R)	GYDRDDKEAPDSDAEGYVEGLDDEEEDEDEEEYDDDAQVVEDEEDR	X3-like (25.4 ± 1.7%)

[4] ANP32A transcripts are identical between the three goose cell lines (GF-R, GN-R and CCL-169).

**Species (cell-line)**	**aa sequence**	**ANP32A transcript (% ± SD)**
Goose (GN-R)[4]	GYDRDDKEAPDSDAEGYVEGLDDEEEDEDVLSLVKDRDDKEAPDSDAEG	X1-like (65.5 ± 0.5%)
Goose (GN-R)	GYDRDDKEAPDSDAEGYVEGLDDEEEDEDVKDRDDKEAPDSDAEGYVEG	X2-like (6.1 ± 0.2%)
Goose (GN-R)	GYDRDDKEAPDSDAEGYVEGLDDEEEDEDEEEYDDDAQVVEDEEDR	X3-like (28.4 ± 0.5%)
Goose (CCL-169)[4]	GYDRDDKEAPDSDAEGYVEGLDDEEEDEDVLSLVKDRDDKEAPDSDAEG	X1-like (66.6 ± 0.6%)
Goose (CCL-169)	GYDRDDKEAPDSDAEGYVEGLDDEEEDEDVKDRDDKEAPDSDAEGYVEG	X2-like (8.8 ± 0.1%)
Goose (CCL-169)	GYDRDDKEAPDSDAEGYVEGLDDEEEDEDEEEYDDDAQVVEDEEDR	X3-like (24.6 ± 0.7%)
Mute swan (SWD-R)	GYDRDDKEAPDSDAEGYVEGLDDEEEDEDVLSLVKDRDDKEAPDSDAEG	X1-like (69.1 ± 1.6%)
Mute swan (SWD-R)	GYDRDDKEAPDSDAEGYVEGLDDEEEDEDVKDRDDKEAPDSDAEGYVEG	X2-like (7.4 ± 0.5%)
Mute swan (SWD-R)	GYDRDDKEAPDSDAEGYVEGLDDEEEDEDEEEYDDDAQVVEDEEDR	X3-like (23.5 ± 1.9%)
Swallow (CS1T1-R)	GYDRDDKEAPDSDAEGYVEGLDDEEEDEDVLSLVKDRDDKEAPDSDAEG	X1-like (14.0 ± 1.1%)
Swallow (CS1T1-R)	GYDRDDKEAPDSDAEGYVEGLDDEEEDEDVKDRDDKEAPDSDAEGYVEG	X2-like (25.2 ± 1.1%)
Swallow (CS1T1-R)	GYDRDDKEAPDSDAEGYVEGLDDEEEDEDEEEYDDDAQVVEDEEDR	X3-like (60.8 ± 2.0%)
Blackbird (TME-R)	GYDRDDKEAPDSDAEGYVEGLDDEEEDEDALSLVKDRDDKEAPDSDAEG	X1-like (36.9 ± 2.0%)
Blackbird (TME-R)	GYDRDDKEAPDSDAEGYVEGLDDEEEDEDVKDRDDKEAPDSDAEGYVEG	X2-like (59.0 ± 1.8%)
Blackbird (TME-R)	GYDRDDKEAPDSDAEGYVEGLDDEEEDEDEEEYDDDAQVVEDEEDR	X3-like (4.1 ± 0.2%)
Magpie (EGE-R)	GYDRDDKEAPDSDAEGYVEGLDDEEEDEDVLSLVKDRDDKEAPDSDAEG	X1-like (31.3 ± 0.2%)
Magpie (EGE-R)	GYDRDDKEAPDSDAEGYVEGLDDEEEDEDVKDRDDKEAPDSDAEGYVEG	X2-like (33.0 ± 1.3%)
Magpie (EGE-R)	GYDRDDKEAPDSDAEGYVEGLDDEEEDEDEEEYDDDAQVVEDEEDR	X3-like (35.7 ± 1.4%)

Alignment of the aa sequences of ANP32A X1-like transcripts as found in the analyzed species:

```
Chicken    GYDRDDKEAPDSDAEGYVEGLDDEEEDEDVLSLVKDRDDKEAP
Duck       GYDRDDKEAPDSDAEGYVEGLDDEEEDEDVLSLVKDRDDKEAP
Mallard    GYDRDDKEAPDSDAEGYVEGLDDEEEDEDVLSLVKDRDDKEAP
Turkey     GYDRDDKEAPDSDAEGYVEGLDDEEEDEDVLSLVKDRDDKEAP
Quail      GYDRDDKEAPDSDAEGYVEGLDDEEEDEDVLSLVKDRDDKEAP
Goose      GYDRDDKEAPDSDAEGYVEGLDDEEEDEDVLSLVKDRDDKEAP
Mute swan  GYDRDDKEAPDSDAEGYVEGLDDEEEDEDVLSLVKDRDDKEAP
Swallow    GYDRDDKEAPDSDAEGYVEGLDDEEEDEDVLSLVKDRDDKEAP
Blackbird  GYDRDDKEAPDSDAEGYVEGLDDEEEDEDALSLVKDRDDKEAP
Magpie     GYDRDDKEAPDSDAEGYVEGLDDEEEDEDVLSLVKDRDDKEAP
           *****************************.*************
```

Alignment of the aa sequences of ANP32A X2-like transcripts:

```
Chicken    GYDRDDKEAPDSDAEGYVEGLDDEEEDEDVKDRDDKEAPDSDAE
Duck       GYDRDDKEAPDSDAEGYVEGLDDEEEDEDVKDRDDKEAPDSDAE
Mallard    GYDRDDKEAPDSDAEGYVEGLDDEEEDEDVKDRDDKEAPDSDAE
Turkey     GYDRDDKEAPDSDAEGYVEGLDDEEEDEDVKDRDDKEAPDSDAE
Quail      GYDRDDKEAPDSDAEGYVEGLDDEEEDEDVKDRDDKEAPDSDAE
Goose      GYDRDDKEAPDSDAEGYVEGLDDEEEDEDVKDRDDKEAPDSDAE
Mute swan  GYDRDDKEAPDSDAEGYVEGLDDEEEDEDVKDRDDKEAPDSDAE
Swallow    GYDRDDKEAPDSDAEGYVEGLDDEEEDEDVKDRDDKEAPDSDAE
Blackbird  GYDRDDKEAPDSDAEGYVEGLDDEEEDEDVKDRDDKEAPDSDAE
Magpie     GYDRDDKEAPDSDAEGYVEGLDDEEEDEDVKDRDDKEAPDSDAE
           ********************************************
```

Alignment of the aa sequences of ANP32A X3-like transcripts:

```
Human      GYDRDDKEAPDSDAEGYVEGLDDEEEDEDEEEYDEDAQVV
Pig        GYDRDDKEASDSDAEGYVEGLDDDEEDEDEEEYDEDAQVV
Dog        GYDRDDKEAPDSDAEGYVEGLDDDEEDEDEEEYDEDAQVV
Mouse      GYDRDNKEAPDSDVEGYVEDD--DEEDEDEEEYDEYAQLV
Bat        GYDRDDKEAPDSDAEGYVEGLDDDEEDEDEEEYDEDAQVV
Horse      GYDRDDKEAPDSDAEGYVEGLDDDEEDEDEEEYDEDAQVV
Chicken    GYDRDDKEAPDSDAEGYVEGLDDEEEDEDEEEYDDDAQVV
Duck       GYDRDDKEAPDSDAEGYVEGLDDEEEDEDEEEYDDDAQDR
Mallard    GYDRDDKEAPDSDAEGYVEGLDDEEEDEDEEEYDDDAQDR
Turkey     GYDRDDKEAPDSDAEGYVEGLDDEEEDEDEEEYDDDAQVV
Goose      GYDRDDKEAPDSDAEGYVEGLDDEEEDEDEEEYDDDAQVV
Mute swan  GYDRDDKEAPDSDAEGYVEGLDDEEEDEDEEEYDDDAQVV
Swallow    GYDRDDKEAPDSDAEGYVEGLDDEEEDEDEEEYDDDAQVV
Blackbird  GYDRDDKEAPDSDAEGYVEGLDDEEEDEDEEEYDDDAQVV
Magpie     GYDRDDKEAPDSDAEGYVEGLDDEEEDEDEEEYDDDAQVV
           *****:*** ***.*****.   :**********: **
```

In bold, the aspartate (**D**) at the 5' end of the splicing site of avian ANP32A. Underlined, the SLS site present in the X1-like variants.

Supplementary Table 2

Summary of ANP32A transcript analyses from different primary chicken tissues

	**ANP32A transcript variant**		
**Tissue**	**X1 (%)**	**X2 (%)**	**X3 (%)**
Brain	74.3	13.3	12.4
Cecum	72.1	16.1	11.8
Eye	71.1	17.3	11.6
Heart	74.1	12.9	13.0
Kidney	71.8	16.6	11.6
Liver	70.5	17.6	11.9
Lung	68.7	18.6	12.8
Small intestine	75.4	13.5	11.1
Skeletal muscle	73.0	17.0	10.0
Spleen	68.3	20.4	11.4

Supplementary Table 3

Summary of cells and cell-lines used in this study

Cell ID	Source	Provider	Species
A549	Human lung epithelial carcinoma	ATCC[1]	*H. sapiens*
BEPC	Human primary bronchial epithelial cells	PromoCell[2]	*H. sapiens*
NEPC	Human primary nasal epithelial cells	PromoCell	*H. sapiens*
SAEPC	Human primary small airway epithelial cells	PromoCell	*H. sapiens*
HTEpC	Human primary tracheal epithelial cells	PromoCell	*H. sapiens*
HEK293T	Human, embryonic kidney (transformed, epithelial)	ATCC	*H. sapiens*
PK-15	Swine kidney adult (spontaneously immortalized, epithelial)	*Lanz et al.*[3]	*S. scrofa*
NSK	Swine kidney newborn (spontaneously immortalized, epithelial)	*Lanz et al.*	*S. scrofa*
MDCK	Canine kidney adult (spontaneously immortalized, epithelial)	ATCC	*C. familiaris*
MEF	Murine embryonic fibroblasts (immortalized)	ATCC	*M. musculus*
PaKi	Pteropid bat kidney (immortalized, epithelial)	*Crameri et al.*[4]	*P. alecto*
NBL6	Horse, dermal fibroblasts	ATCC	*E. caballus*
DF-1	Chicken, embryonal fibroblasts spontaneously transformed	ATCC	*G. gallus*
LMH-1	Chicken, hepatocellular carcinoma (chemically induced, epithelial)	ATCC	*G. gallus*
SL-29	Chicken, embryonal fibroblasts	ATCC	*G. gallus*
CAM	Chicken, chorioallantoic membrane	AnimalCo	*G. gallus*
CCL-141	Duck, embryonic fibroblasts	ATCC	*A. platyrhynchus dom.*
SEF-R	Mallard, whole embryo	CCLV FLI[5]	*A. platyrhynchos*
PF-R	Turkey, whole embryo	CCLV FLI	*M. gallopavo f. dom.*
QT-6	Quail, methylcholanthrene induced fibroblast	CCLV FLI	*C. coturnix japonica*
SMF-R	Gull, whole Embryo	CCLV FLI	*L. canus*
GF-R	Goose, whole embryo	CCLV FLI	*A. anser f. dom.*
GN-R	Goose, kidney adult(epithelial)	CCLV FLI	*A. anser f. dom.*
CCL-169	Goose, sternum fibroblasts	ATCC	*A. anser*
SWD-R	Mute swan, intestine (epithelial)	CCLV FLI	*C. olor*
CS1T1-R	Swallow, whole embryo	CCLV FLI	*P. pyrrhonota*
TME-R	Blackbird, whole embryo	CCLV FLI	*T. merula*
EGE-R	Magpie, whole embryo	CCLV FLI	*P. pica*

[1] ATCC, American Type Culture Collection.
[2] PromoCell GmbH (Heidelberg, Germany)
[3] Lanz, C., Yanguez, E., Andenmatten, D., and Stertz, S. (2015). Swine interferon-inducible transmembrane proteins potently inhibit influenza A virus replication. J Virol *89*, 863-869.
[4] Crameri, G., Todd, S., Grimley, S., McEachern, J.A., Marsh, G.A., Smith, C., Tachedjian, M., De Jong, C., Virtue, E.R., Yu, M., *et al.* (2009). Establishment, immortalisation and characterisation of pteropid bat cell lines. PLoS One *4*, e8266.
[5] Collection of Cell Lines in Veterinary Medicine (CCLV), Friedrich-Loeffler-Institut (FLI), Insel Riems, Germany.

Supplementary Table 4

Summary of primer pairs used for the NGS-based determination of ANP32A splice variants in different species

Primer ID	Sequence	Species
humANP32A-NGS-F	CTT TCC CTA CAC GAC GCT CTT CCG ATC TTA TCT CGA CGG CTA TGA CC[1]	Human (*H. sapiens*)
humANP32A-NGS-R	GAC TGG AGT TCA GAC GTG TGC TCT TCC GAT CTA TCG TTA TAA CCT TCT TCA TCC	Human (*H. sapiens*)
ssANP32_NGS-F	CTT TCC CTA CAC GAC GCT CTT CCG ATC TTA TCT CGA TGG CTA CGA CC	Pig (*S. scrofa*)
ssANP32A_NGS-R	GAC TGG AGT TCA GAC GTG TGC TCT TCC GAT CTA TCG TTA TAA CCT TCC TCA TCC	Pig (*S. scrofa*)
clANP32A_NGS-R	CTT TCC CTA CAC GAC GCT CTT CCG ATC TTA TCT CGA TGG CTA CGA CC	Dog (*C. familiaris*)
clANP32A_NGS-R	GAC TGG AGT TCA GAC GTG TGC TCT TCC GAT CTT TAT AAC CCT CTT CAT CCT CC	Dog (*C. familiaris*)
mmANP32A_NGS-F	CTT TCC CTA CAC GAC GCT CTT CCG ATC TTA CCT CGA TGG CTA TGA CAG	Mouse (*M. musculus*)
mmANP32A_NGS-R	GAC TGG AGT TCA GAC GTG TGC TCT TCC GAT CTT CAT TGT AAC CTT CCT CAT CC	Mouse (*M. musculus*)
batANP32A_NGS-F	CTT TCC CTA CAC GAC GCT CTT CCG ATC TTG ATG GCT ATG ACC GGG ATG	Bat (*P. alecto*)
batANP32A_NGS-R	GAC TGG AGT TCA GAC GTG TGC TCT TCC GAT CTA CTT CCC CGT CGT TAT AAC C	Bat (*P. alecto*)
ecANP32A_NGS-F	CTT TCC CTA CAC GAC GCT CTT CCG ATC TTA CCT CGA CGG CTA CGA CC	Horse (*E. caballus*)
ecANP32A_NGS-R	GAC TGG AGT TCA GAC GTG TGC TCT TCC GAT CTA TCT ACT TCC CCG TCA TTA TAA CC	Horse (*E. caballus*)
ggANP32A_NGS-F	CTT TCC CTA CAC GAC GCT CTT CCG ATC TAT GGC TAC GAT CGG GAT GAC	Chicken (*G. gallus*)
ggANP32A_NGS-R	GAC TGG AGT TCA GAC GTG TGC TCT TCC GAT CTT CTT CAT CTT CTA CTA CCT GAG C	Chicken (*G. gallus*)
apANP32A-NGS-2-F	CTT TCC CTA CAC GAC GCT CTT CCG ATC TCA ACT CAC ATA CCT CGA TGG	Duck (*A. platyrhynchus dom.*)
apANP32A-NGS-2-R	GAC TGG AGT TCA GAC GTG TGC TCT TCC GAT CTT GAG CGT CAT CAT CAT ACT CC	Duck (*A. platyrhynchus dom.*)
apANP32A_NGS-2-F	CTT TCC CTA CAC GAC GCT CTT CCG ATC TCA ACT CAC ATA CCT CGA TGG	Mallard (*A. platyrhynchos*)
ansANP32A_NGS-R	GAC TGG AGT TCA GAC GTG TGC TCT TCC GAT CTT CTT CGT CTT CTA CTA CCT GAG C	Mallard (*A. platyrhynchos*)
ggANP32A_NGS-F	CTT TCC CTA CAC GAC GCT CTT CCG ATC TAT GGC TAC GAT CGG GAT GAC	Turkey (*M. gallopavo*)
ggANP32A_NGS-R	GAC TGG AGT TCA GAC GTG TGC TCT TCC GAT CTT CTT CAT CTT CTA CTA CCT GAG C	Turkey (*M. gallopavo*)
cjANP32A_NGS-F	CTT TCC CTA CAC GAC GCT CTT CCG ATC TAT ACC TCG ATG GCT ACG ATC GG	Quail (*Coturnix c. japonica*)
cjANP32A_NGS-R	GAC TGG AGT TCA GAC GTG TGC TCT TCC GAT CTA CGT CAC CAT CAT TAT AGC CTT CC	Quail (*Coturnix c. japonica*)

[1] Underlined, the priming sites for 2nd round PCR primers, TS-D50x+TS-D70y of the Illumina®, TruSeq HT series.

ruffANP32A-NGS-F	CTT TCC CTA CAC GAC GCT CTT CCG ATC TAT GGC TAC GAC CGG GAC GAC	Common gull (*L. canus*)
ansANP32A_NGS-R	GAC TGG AGT TCA GAC GTG TGC TCT TCC GAT CTT CTT CGT CTT CTA CTA CCT GAG C	Common gull (*L. canus*)
ansANP32A_NGS-F	CTT TCC CTA CAC GAC GCT CTT CCG ATC TAT GGC TAC GAT CGG GAC GAC	Goose (*Anser anser f. dom.*)
ansANP32A_NGS-R	GAC TGG AGT TCA GAC GTG TGC TCT TCC GAT CTT CTT CGT CTT CTA CTA CCT GAG C	Goose (*Anser anser f. dom.*)
apANP32A-NGS-2-F	CTT TCC CTA CAC GAC GCT CTT CCG ATC TCA ACT CAC ATA CCT CGA TGG	Mute swan (*C. olor*)
ansANP32A_NGS-R	GAC TGG AGT TCA GAC GTG TGC TCT TCC GAT CTT CTT CGT CTT CTA CTA CCT GAG C	Mute swan (*C. olor*)
starlANP32A-NGS-F	CTT TCC CTA CAC GAC GCT CTT CCG ATC TAC GGA TAC GAC CGG GAT GAC	Swallow (*P. pyrrhonota*)
starlANP32A-NGS-R	GAC TGG AGT TCA GAC GTG TGC TCT TCC GAT CTT CTT CAT CCT CCA CTA CCT GAG C	Swallow (*P. pyrrhonota*)
starlANP32A-NGS-F	CTT TCC CTA CAC GAC GCT CTT CCG ATC TAC GGA TAC GAC CGG GAT GAC	Blackbird (*T. merula*)
starlANP32A-NGS-R	GAC TGG AGT TCA GAC GTG TGC TCT TCC GAT CTT CTT CAT CCT CCA CTA CCT GAG C	Blackbird (*T. merula*)
corvusANP32-NGS-F	CTT TCC CTA CAC GAC GCT CTT CCG ATC TAT GGA TAC GAC CGG GAT GAC	Magpie (*P. pica*)
corvusANP32-NGS-R	GAC TGG AGT TCA GAC GTG TGC TCT TCC GAT CTT CCT CGT CCT CCA CTA CCT GAG C	Magpie (*P. pica*)
3xFl-ANP32A-NGS-F	CTT TCC CTA CAC GAC GCT CTT CCG ATC TAC GGC TAC GAC CGG GAC GAC	Exogenous ANP32A
3xFl-ANP32A-NGS-R	GAC TGG AGT TCA GAC GTG TGC TCT TCC GAT CTT CTT CAT CTT CCA CCA CCT GGG C	Exogenous ANP32A
