# Supplementary material for: Profiling host ANP32A splicing landscapes to predict influenza A virus polymerase adaptation
Source: Nat Commun. 2019 Jul 30;10:3396. doi: 10.1038/s41467-019-11388-2 (PMC6667478; doi:10.1038/s41467-019-11388-2)
Supplement: Supplementary file 3 — Reporting Summary [file 41467_2019_11388_MOESM3_ESM.pdf]

nature research

Corresponding author(s): Benjamin G. Hale

Last updated by author(s): 2019/06/04

Reporting Summary

Nature Research wishes to improve the reproducibility of the work that we publish. This form provides structure for consistency and transparency in reporting. For further information on Nature Research policies, see Authors & Referees and the Editorial Policy Checklist.

Statistics

For all statistical analyses, confirm that the following items are present in the figure legend, table legend, main text, or Methods section.

n/a	Confirmed	
☐	☒	The exact sample size (*n*) for each experimental group/condition, given as a discrete number and unit of measurement
☐	☒	A statement on whether measurements were taken from distinct samples or whether the same sample was measured repeatedly
☐	☒	The statistical test(s) used AND whether they are one- or two-sided<br>*Only common tests should be described solely by name; describe more complex techniques in the Methods section.*
☒	☐	A description of all covariates tested
☐	☒	A description of any assumptions or corrections, such as tests of normality and adjustment for multiple comparisons
☐	☒	A full description of the statistical parameters including central tendency (e.g. means) or other basic estimates (e.g. regression coefficient) AND variation (e.g. standard deviation) or associated estimates of uncertainty (e.g. confidence intervals)
☒	☐	For null hypothesis testing, the test statistic (e.g. *F*, *t*, *r*) with confidence intervals, effect sizes, degrees of freedom and *P* value noted<br>*Give P values as exact values whenever suitable.*
☒	☐	For Bayesian analysis, information on the choice of priors and Markov chain Monte Carlo settings
☒	☐	For hierarchical and complex designs, identification of the appropriate level for tests and full reporting of outcomes
☒	☐	Estimates of effect sizes (e.g. Cohen's *d*, Pearson's *r*), indicating how they were calculated

Our web collection on statistics for biologists contains articles on many of the points above.

Software and code

Policy information about availability of computer code

Data collection	No code was used to collect data in this study
Data analysis	The customized codes and mathematical algorithms described in this paper can be freely accessed at https://github.com/magnuscar/FluAdaptation. In addition, R package DescTools, BEAST2 and GraphPad Prism 7.03 were used.

For manuscripts utilizing custom algorithms or software that are central to the research but not yet described in published literature, software must be made available to editors/reviewers. We strongly encourage code deposition in a community repository (e.g. GitHub). See the Nature Research guidelines for submitting code & software for further information.

Data

Policy information about availability of data

All manuscripts must include a data availability statement. This statement should provide the following information, where applicable:

- Accession codes, unique identifiers, or web links for publicly available datasets
- A list of figures that have associated raw data
- A description of any restrictions on data availability

The authors declare that all data supporting the findings of this study are available within the paper and its supplementary information files. The source data for indicated figures are provided as a Source Data file.

Field-specific reporting

Please select the one below that is the best fit for your research. If you are not sure, read the appropriate sections before making your selection.

☒ Life sciences ☐ Behavioural & social sciences ☐ Ecological, evolutionary & environmental sciences

For a reference copy of the document with all sections, see nature.com/documents/nr-reporting-summary-flat.pdf

Life sciences study design

All studies must disclose on these points even when the disclosure is negative.

Sample size	No sample-size calculations were performed. Sample size was determined to be adequate based on the magnitude and consistency of measurable differences between groups.
Data exclusions	No data were excluded from analyses.
Replication	Most experiments were repeated 3 independent times. The number of repeats for each experiment is reported in the figure legends.
Randomization	No randomization methods were used for the experimental work because this was not applicable to the types of experiments performed.
Blinding	Investigators were not blinded.

Reporting for specific materials, systems and methods

We require information from authors about some types of materials, experimental systems and methods used in many studies. Here, indicate whether each material, system or method listed is relevant to your study. If you are not sure if a list item applies to your research, read the appropriate section before selecting a response.

Materials & experimental systems

n/a	Involved in the study	
☐	☒	Antibodies
☐	☒	Eukaryotic cell lines
☒	☐	Palaeontology
☒	☐	Animals and other organisms
☒	☐	Human research participants
☒	☐	Clinical data

Methods

n/a	Involved in the study	
☒	☐	ChIP-seq
☒	☐	Flow cytometry
☒	☐	MRI-based neuroimaging

Antibodies

Antibodies used	FLAG M2 antibody F1804 (Sigma), a custom rabbit PB2 polyclonal anti-serum, PA antibody GTX118991 (Genetex), actin antibody A2103 (Sigma), ANP32A antibody ab51013 (Abcam), and ANP32B antibody ab184565 (Abcam).
Validation	No additional validation was performed beyond the manufacturer's data available from the appropriate website. For the influenza A virus proteins (PB2 and PA), as well as FLAG-tagged proteins, western blot reactivity was not observed unless the appropriate construct was transfected into human 293T cells. The band recognized by the ANP32A antibody was not detectable in human ANP32A-KO A549 cells (see manuscript).

Eukaryotic cell lines

Policy information about cell lines

Cell line source(s)	Sources for all cell-lines are detailed in Supplementary Table 3.
Authentication	No cell-line was explicitly authenticated, although during the NGS process, cell-line unique ANP32A sequences were generated that matched the appropriate species following BLAST analysis.
Mycoplasma contamination	Cell lines used in the Hale group are routinely tested for mycoplasma contamination by sending representative samples for mycoplasma testing to GATC, Germany. None of the used cell lines ever tested positive for mycoplasma.
Commonly misidentified lines (See ICLAC register)	None used.
